# Occlusion haute révélant une pneumatose kystique diffuse du grêle

**DOI:** 10.11604/pamj.2021.38.297.27929

**Published:** 2021-03-22

**Authors:** Mohammed Najih, Aziz Zentar

**Affiliations:** 1 Service de Chirurgie Viscérale, Hôpital Militaire d’Instruction Mohammed V, Rabat, Maroc

**Keywords:** Pneumatose kystique, occlusion, approche chirurgicale, Pneumatosis cystoides intestinalis, occlusion, surgical approach

## Abstract

Pneumatosis cystoides intestinalis is a rare disorder characterized by the presence of gas-filled cysts in the intestinal wall. It can involve the whole digestive tract, in particular the small intestine and the colon. Diagnosis is based on computed tomography (CT) scan, surgery is performed in patients with complications. We here report a rare case of diffuse pneumatosis cystoides intestinalis resulting in occlusive events requiring surgery. This consisted of resection of proximal small intestine (120 cm) followed by termino-terminal microsurgical anastomosis 5 cm from the duodeno-jejunal flexure.

## Image en médecine

La pneumatose kystique intestinale est une affection rare caractérisée par la présence de kystes gazeux dans la paroi intestinale, pouvant atteindre l'ensemble du tube digestif, avec une prédilection pour l'intestin grêle et le côlon. Le diagnostic est évoqué sur le scanner, l'intervention chirurgicale est indiquée en cas de complications. Nous rapportons une observation rare de pneumatose kystique étendue du grêle, source de manifestations occlusives imposant l'intervention chirurgicale qui consistait en une résection de 120cm du grêle proximal suivie d´une anastomose termino-terminale à 5cm de l´angle duodéno-jéjunal.

**Figure 1:**
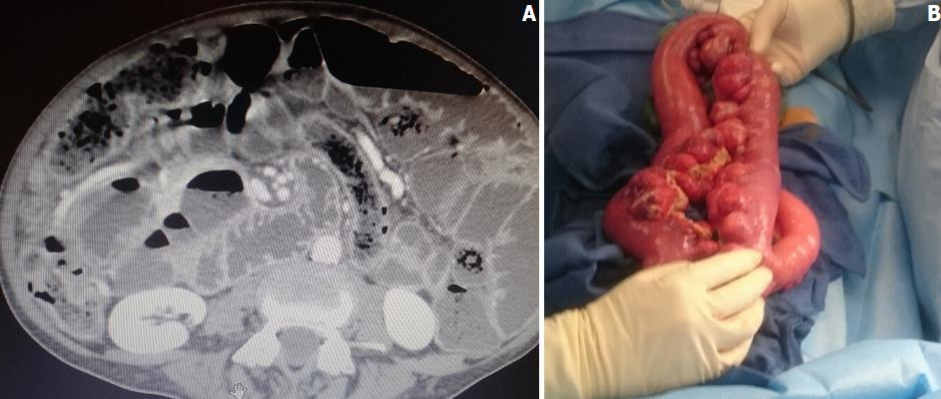
A) tomodensitométrie abdominale, coupe axiale montrant une occlusion sur pneumatose kystique du grêle; B) image peropératoire montrant la pneumatose kystique étendue du grêle

